# The Behavioral and Neuroinflammatory Impact of Ketamine in a Murine Model of Depression and Liver Damage

**DOI:** 10.3390/ijms26083558

**Published:** 2025-04-10

**Authors:** Mădălina Iuliana Mușat, Ana-Maria Ifrim-Predoi, Smaranda Ioana Mitran, Eugen Osiac, Bogdan Cătălin

**Affiliations:** 1 Experimental Research Centre for Normal and Pathological Aging, University of Medicine and Pharmacy of Craiova, 200349 Craiova, Romania; madalina.musat3@gmail.com (M.I.M.); bogdan.catalin@umfcv.ro (B.C.); 2 Department of Surgery, University of Medicine and Pharmacy of Craiova, 200349 Craiova, Romania; anamaria.ifrim@umfcv.ro; 3 Department of Physiology, University of Medicine and Pharmacy of Craiova, 200349 Craiova, Romania; 4 Department of Biophysics, University of Medicine and Pharmacy, 200349 Craiova, Romania

**Keywords:** depression, CUMS, NAFLD, MCD diet, ketamine, behavior

## Abstract

Non-alcoholic fatty liver disease (NAFLD) has been associated with depression and inadequate response to antidepressants. While ketamine has demonstrated efficacy in treating depression, its impact on pre-existing liver injury and depression remains unclear. This study aimed to evaluate the effects of ketamine treatment in a murine model of depression and liver damage, considering age-related differences. Young and aged male C57BL/6N mice were submitted to chronic unpredictable mild stress (CUMS) and methionine–choline-deficient (MCD) diet to induce depressive-like behavior and NAFLD. Behavioral testing (sucrose preference test, open field test, novel object recognition test, Crawley’s sociability test) were used to assess ketamine’s (50 mg/kg) effect on behavior. Hepatic ultrasonography was utilized to evaluate liver status. The cortical and hippocampal NeuN+, GFAP+, and Iba1+ signals were quantified for each animal. Ketamine administration proved effective in relieving anhedonia and anxiety-like behavior, regardless of liver damage. Although ketamine treatment did not improve memory in animals with liver damage, it enhanced sociability, particularly in aged subjects. The acute administration of ketamine did not affect the severity of liver injury, but seems to affect astrogliosis and neuronal loss. Although animal models of depression only replicate certain clinical features of the condition, they remain valuable for evaluating the complex and varied effects of ketamine. By applying such models, we could demonstrate ketamine’s therapeutic versatility, and also indicate that responses to the treatment may differ across different age groups.

## 1. Introduction

As life expectancy increases, the additional stress of modern living can generate unexpected health problems. One notable example is a sudden rise in the prevalence of depression [1,2], with estimations indicating that at least one in five individuals may develop depression at some point in their lifetime [3]. While this increased incidence is concerning, what makes it even more critical is the lack of information regarding treatment strategies for patients with depression associated with various other chronic diseases [4,5,6]. This is concerning for depressed patients, as chronic associated pathologies are known for worsening outcome factors [7,8].

Recently, one such association has been reported between depression and non-alcoholic fatty liver disease (NAFLD) [9]. This particular association seems to be of note as populational studies have shown an increase in the prevalence of NAFLD itself and its association with depression [10]. While the exact mechanisms linking depression and NAFLD remain unclear [8], various studies have reported results ranging from a modest [11] to a strong association between the two [10,12], with evidence also suggesting a potential bidirectional association [9,13]. Understanding and addressing this relationship is crucial for developing effective preventive and therapeutic strategies. However, treating depression may become challenging in the presence of NAFLD, as most antidepressants are metabolized by the liver; thus, the administration of antidepressants can pose a higher risk in individuals with NAFLD [14,15]. One existing concern is that antidepressant medication can interfere with liver enzymes involved in drug metabolism, potentially leading to hepatotoxicity or worsening liver function [16]. Furthermore, since patients with depression associated with NAFLD may show an inadequate response to antidepressant treatment [8], the need for alternative pharmacological strategies becomes apparent.

Within these frames, ketamine has emerged as a promising therapeutic agent with multifaceted pharmacological properties [17,18]. Ketamine treatment appears to be effective in a multitude of depression forms, including treatment-resistant depression [19,20] and depression with severe suicidal ideation [21]. The positive outcomes reported for this category of patients, for up to six weeks [22], make ketamine more than appealing for health professionals. However, prolonged and/or repeated administration may lead to ketamine-induced liver injury (KILI) [23,24]. While there is growing interest in ketamine as a potential therapy for depression, the impact of ketamine on individuals with pre-existing NAFLD is not extensively studied.

The efficacy of administering ketamine to mice in a single dose of 10 mg/kg for anxiety and depressive-like behaviors seems to be age-dependent, with reports showing that the effectiveness of ketamine in early-life stress may be limited or absent, and the antidepressant effect of ketamine may be dependent on the type of stress applied; thus, ketamine may be ineffective at treating mood disorders while exacerbating long-lasting aggression associated with early-life stress [25]. Additionally, some reports showed that 15 mg/kg acute ketamine exposure can increase anxiety-like behavior, but do not alter object recognition memory, depression-like behavior, or plasma corticosterone [26]. Thus, while low doses of 10–15 mg/kg are generally regarded as standard and often appropriate, their effectiveness has not been fully established, and may vary in particular situations.

This study aimed to examine the effects of ketamine in animals exhibiting depressive-like behavior with liver damage, focusing on age-specific effects and the impact of neuroinflammatory responses—a scenario that, to the best of our knowledge, has not been previously explored. Furthermore, we aimed to investigate whether the antidepressant effect could be prolonged at a higher dose and to evaluate whether such a dose might exacerbate liver damage in animals with pre-existing liver injury, enabling us to assess potential additional consequences.

## 2. Results

### 2.1. Acute Administration of Ketamine Does Not Affect the Severity of Liver Injury

**Figure 1:**
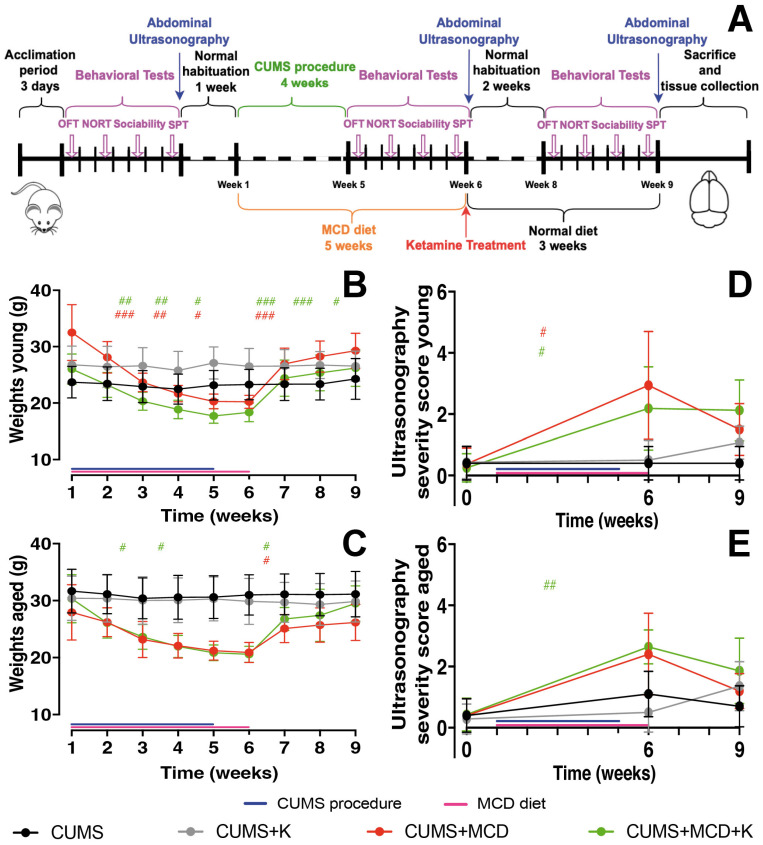
The weekly experimental design of the present study included a 3-day acclimatization period, followed by one week of behavioral testing and abdominal ultrasonography, and then one week of normal habituation before the initiation of the CUMS protocol and MCD diet. In week 5, the behavioral and imaging assessments were repeated, and the ketamine treatment was administered at the beginning of week 6. All measurements were repeated two weeks later, in week 8. (**A**). Body weights and ultrasonography severity scores (**B**–**E**). The graphs show mean values ± SD, # *p* < 0.05, ## *p* < 0.01, and ### *p* < 0.001 displaying differences between sessions.

The two-way ANOVA performed on the young animals’ weight, revealed variances between Sessions (F_2.334,56.00_ = 78.76, *p* < 0.0001) and Treatments (F_3,24_ = 4.597, *p* = 0.0111). Significant interaction was also noticed between sessions and treatments (F_24,192_ = 24.06, *p* < 0.0001). Post hoc tests showed that all animals fed an MCD diet experienced a weekly reduction in body mass (Figure 1B). After stopping the MCD diet, CUMS + MCD (*p* = 0.0003) and CUMS + MCD + K (*p* = 0.0008) animals increased their body weights. The CUMS + MCD + K group maintained this increase in body weight until the end of the experiment (Figure 1B). A post hoc test revealed no differences between treatments at the end of the experiment (*p* > 0.05). However, in order to observe how the MCD diet, CUMS protocol, and ketamine treatment affect weight dynamics, some differences after the CUMS procedure and MCD diet have been identified between CUMS and CUMS + MCD + K (*p* = 0.0396) and CUMS + K compared to both CUMS + MCD (*p* = 0.0057) and CUMS + MCD + K (*p* = 0.0009) (Figure 1B).

In aged animals, the two-way ANOVA showed differences between sessions (F_2.879,57.58_ = 32.29, *p* < 0.0001), treatments (F_3,20_ = 6.575, *p* = 0.0028) and interaction (F_24,160_ = 11.19, *p* < 0.0001). A post hoc test revealed a weekly decrease in body mass in MCD-fed animals (Figure 1C), and a consecutive weight gain at the end of MCD diet for CUMS + MCD (*p* = 0.0266) and CUMS + MCD + K (*p* = 0.0134) groups. No differences between treatments were observed (*p* > 0.05) at the end of the experiment. However, after MCD diet and CUMS procedure, a post hoc test showed differences between CUMS and CUMS + MCD (*p* = 0.0054), CUMS + MCD + K (*p* = 0.0062), and CUMS + K compared to CUMS + MCD (*p* = 0.0028) and CUMS + MCD + K (*p* = 0.0025) (Figure 1C).

The ultrasonography performed after the MCD diet was able to confirm the presence of liver damage in all young animals fed the MCD diet (Figure 1D). Non-parametric Kruskal–Wallis test showed increased severity scores for CUMS + MCD (*p* = 0.0200) and CUMS + MCD + K (*p* = 0.0500) before the ketamine administration, compared to the baseline (Figure 1D). No differences in the severity scores between the groups were observed (*p* > 0.05).

Even though all aged animals developed micro- and macro- nodules, from baseline to post-MCD diet, significant differences were observed only in CUMS + MCD + K (*p* = 0.0086) group, while CUMS + MCD animals displayed only a tendency in the ultrasonography severity score without reaching the statistical threshold (*p* > 0.05) (Figure 1E). After MCD diet, CUMS + MCD + K mice displayed increased severity scores compared to CUMS + K group (*p* = 0.0188). No differences in the severity scores between the groups were observed at the end of the experiment (*p* > 0.05).

### 2.2. Ketamine Alleviates Anhedonia and Anxiety-like Behavior, Regardless of Liver Damage

**Figure 2:**
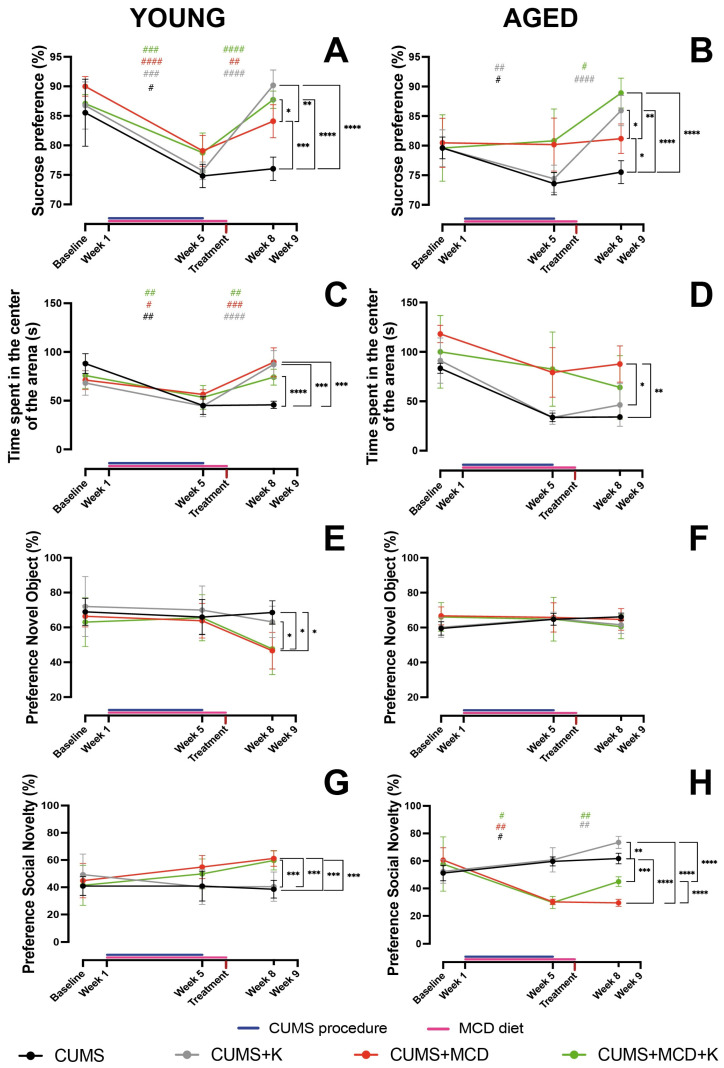
Behavioral assessments. Ketamine treatment led to an increased sucrose preference for (**A**) all young ketamine-treated animals, regardless of liver injury. After ketamine administration, CUMS + K, CUMS + MCD, and CUMS + MCD + K animals exhibited increased sucrose preference compared to CUMS mice. In (**B**) aged animals, SPT revealed increased sucrose preference for ketamine-treated mice, regardless of the diet, with the CUMS + MCD + K group presenting the highest preference index (88.90 ± 2.52%). OFT performed in (**C**) young animals showed that both ketamine and normal diets led to decreased anxiety-like behavior. Conversely, in (**D**) aged mice, the CUMS + MCD group displayed decreased anxiety-like behavior compared to CUMS + K and CUMS animals. The NORT of (**E**) young CUMS + MCD and CUMS + MCD + K mice revealed decreased preference for novel object compared to CUMS animals. No differences were observed in (**F**) aged animals. Crawley’s sociability test revealed decreased preference for social novelty for (**G**) young CUMS mice compared to CUMS + MCD and CUMS + MCD + K. In (**H**) aged animals, sociability increased after ketamine treatment, regardless of diet, with CUMS + K displaying the highest preference index (73.46 ± 4.45%). The graphs show mean values ± SD, * *p* < 0.05, ** *p* < 0.01, *** *p* < 0.001 and **** *p* < 0.0001 displaying differences between treatments, and # *p* < 0.05, ## *p* < 0.01, ### *p* < 0.001 and #### *p* < 0.0001 displaying differences between sessions.

The two-way ANOVA performed in order to assess the preference for sucrose in young animals showed differences between treatments (F_3,24_ = 10.60, *p* = 0.0001) and sessions (F_1.723,41.36_ = 143.6, *p* < 0.0001), with significant interaction between the analyzed factors (F_6,48_ = 13.73, *p* < 0.0001). Post hoc test revealed that all young mice exhibited a decrease in the sucrose preference after CUMS procedure, compared to baseline. Ketamine treatment led to an increased intake for all treated animals, regardless of liver injury (*p* > 0.001). Interestingly, the CUMS + MCD group also exhibited an increase sucrose preference, following the animals’ transition to a normal diet (*p* = 0.0077) (Figure 2A) (Supplementary Table S1). After ketamine treatment, young CUMS + K (90.18 ± 2.61%) (*p* < 0.0001), CUMS + MCD (84.10 ± 2.80%) (*p* = 0.0005), and CUMS + MCD + K (87.73 ± 1.45%) (*p* < 0.0001) groups showed increased sucrose preference compared to CUMS mice (76.05 ± 1.99%). Additional differences were also observed between CUMS + MCD compared to CUMS + K (*p* = 0.0039) and CUMS + MCD + K (*p* = 0.0353) (Figure 2A).

In aged animals, two-way ANOVA also revealed variances in SPT between treatments (F_3,20_ = 7.181, *p* = 0.0019), sessions (F_1.406,28.12_ = 20.50, *p* < 0.0001), with significant interaction between the analyzed factors (F_6,40_ = 7.974, *p* < 0.0001). A post hoc test performed between baseline and after CUMS and liver injury induction revealed differences in aged animals subjected to CUMS, with no differences between MCD-fed mice (*p* > 0.05). Ketamine-treated animals showed increased sucrose preference, regardless of the diet (Figure 2B) (Supplementary Table S1). SPT performed after treatment revealed an increased sucrose preference in CUMS + K (85.93 ± 2.56%) (*p* < 0.0001), CUMS + MCD (81.17 ± 2.49%) (*p* = 0.0184), and CUMS + MCD + K (88.90 ± 2.52%) (*p* < 0.0001) compared to CUMS mice (75.51 ± 1.94%). Also, aged CUMS + K (*p* = 0.0435) and CUMS + MCD + K (*p*=0.0025) mice exhibited increased sucrose preference compared to the CUMS + MCD group (Figure 2B).

For the OFT performed in young animals, the two-way ANOVA showed significant differences in treatments (F_3,24_ = 3.296, *p* = 0.0377), sessions (F_1.764,42.33_ = 53.43, *p* < 0.0001), with significant interaction between the analyzed factors (F_6,48_ = 11.71, *p* < 0.0001). A post hoc test, comparing the effect of MCD and CUMS to baseline, revealed that mice exhibited increased anxiety-like behavior, regardless of diet. The ketamine treatment and normal diet led to a decreased anxiety-like behavior (Figure 2C) (Supplementary Table S2). Young CUMS mice exhibited decreased time in the center of the arena (45.69 ± 3.82 s), compared to CUMS + K (87.11 ± 14.27 s) (*p* = 0.0006), CUMS + MCD (89.56 ± 14.78 s) (*p* = 0.0002), and CUMS + MCD + K (74.13 ± 8.20 s) (*p* > 0.0001) (Figure 2C).

The two-way ANOVA performed on data generated by OFT obtained from aged animals revealed significant variances between treatments (F_3,20_ = 7.431, *p* = 0.0016) and sessions (F_1.410,28.19_ = 36.85, *p* < 0.0001), with no differences between interaction (F_6,40_ = 1.790, *p* = 0.1259). However, when one-way ANOVA was performed on the data obtained from the end of the experiment, differences were revealed between treatments (F_3,20_ = 5.435, *p* = 0.0067). After treatment, aged CUMS + MCD mice displayed increased time in the center of the arena (87. 66 ± 18.37 s) compared to CUMS + K (46.28 ± 21.66) (*p* = 0.0266) and CUMS (34.09 ± 2.79 s) (*p* = 0.0069) (Figure 2D).

### 2.3. Ketamine Does Not Enhance Memory in All Animals with Liver Damage, but It Improves Sociability in Aged Ones

When NORT was performed in young animals, the two-way ANOVA showed variances between treatments (F_3,24_ = 3.857, *p* = 0.0220) and sessions (F_1.808,43.39_ = 7.424, *p* = 0.0023), but no differences in interaction (F_6,48_ = 1.339, *p* = 0.2585). However, when one-way ANOVA was applied on the data obtained from the end of the experiment, it showed differences between treatments (F_3,24_ = 6.421, *p* = 0.0024). Young CUMS mice exhibited an increased preference for novel objects (68.60 ± 6.75%) compared to CUMS + MCD (46.61 ± 10.53%) (*p* = 0.0102) and CUMS + MCD + K mice (47.68 ± 14.76%) (*p* = 0.0151). Also, the CUMS + K group displayed an increased recognition index (63.23 ± 9.05%) compared to CUMS + MCD animals (*p* = 0.0383) (Figure 2E). In aged animals, no significant differences were observed in NORT in any statistical analysis (Figure 2F).

The two-way ANOVA, applied in a Crawley’s sociability test in young animals, revealed variances between treatments (F_3,24_ = 5.623, *p* = 0.0046), but not in sessions (F_1.509,36.21_ = 2.153, *p* = 0.1416). Significant interaction was also noticed (F_6,48_ = 3.183, *p* = 0.0104). One-way ANOVA performed on the data obtained at the end of the experiment revealed differences between treatments (F_3,24_ = 16.68, *p* < 0.0001). After treatment, young CUMS + MCD (61.22 ± 5.80%) (*p* = 0.0002) and CUMS + MCD + K (59.67 ± 6.92%) (*p* = 0.0004) mice exhibited increased preference for social novelty, in comparation with the CUMS group (38.55 ± 6.50%). Similarly, CUMS + MCD (*p* = 0.0001) and CUMS + MCD + K animals (*p* = 0.0004) showed an increased index compared to the CUMS + K group (40.45 ± 10.73%) (Figure 2G).

In the sociability test performed in aged animals, two-way ANOVA showed significant differences in treatments (F_3,20_ = 25.84, *p* < 0.0001), sessions (F_1.267,25.35_ = 10.10, *p* = 0.0022), and interaction (F_6,40_ = 16.21, *p* < 0.0001). A post hoc test, comparing mice with liver injury and subjected to CUMS, revealed that animals fed an MCD diet exhibited a decrease in the preference for social novelty. The preference increased after ketamine treatment, regardless of diet (Figure 2H) (Supplementary Table S2). At the end of the experiment, aged CUMS (61.76 ± 3.83%) (*p* < 0.0001), CUMS + K (73.46 ± 4.45%) (*p* < 0.0001), and CUMS + MCD + K (44.96 ± 3.50%) (*p* < 0.0001) animals showed an increased preference for social novelty compared to the CUMS + MCD group (29.57 ± 2.60%). CUMS (*p* = 0.0002) and CUMS + K (*p* < 0.0001) mice also displayed an increased social preference compared to CUMS + MCD + K. Ketamine treatment increased the preference index in CUMS animals fed a normal diet, compared to untreated ones (*p* = 0.0034) (Figure 2H).

### 2.4. Ketamine Is More Effective in Young Compared to Aged Animals

**Figure 3:**
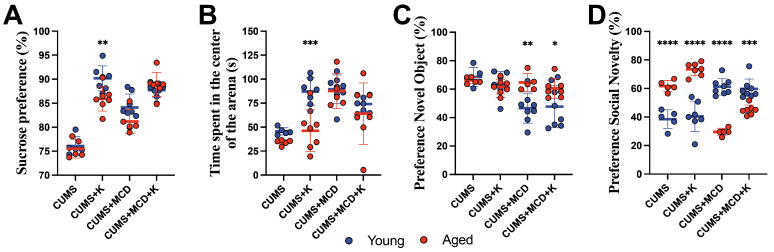
Age-related differences in behavioral tests. (**A**) The SPT performed after treatment and normal food revealed increased sucrose preference in young CUMS + K mice compared to aged ones. Young CUMS + K mice also exhibited reduced anxiety-like behavior in (**B**) OFT when compared to aged counterparts. Regardless of treatment administration, all young MCD-fed mice displayed a decreased preference for novel objects in (**C**) NORT at the end of the experiment. In a (**D**) social novelty test, aged mice fed a normal diet and submitted to the CUMS procedure showed increased preference for social novelty compared to young groups, and MCD food led to decreased index in all aged, compared to young, animals, regardless of treatment. The graphs show mean values ± SD, * *p* < 0.05, ** *p* < 0.01, *** *p* < 0.001 and **** *p* < 0.0001.

Assessing the differences in anhedonia between young and aged animals using two-way ANOVA two weeks after ketamine treatment revealed variances between treatments (F_3,44_ = 71.80, *p* < 0.0001) and age (F_1,44_ = 6.024, *p* = 0.0181), with significant interaction between the tested factors (F_3,44_ = 3.691, *p* = 0.0187). A post hoc test showed that young CUMS + K mice displayed an increase in sucrose preference (90.18 ± 2.61%) compared to aged ones (86.93 ± 2.56%) (*p* = 0.0062) (Figure 3A). No differences were observed between CUMS and MCD groups, regardless of age (*p* > 0.05).

In OFT, the two-way ANOVA showed differences between treatments (F_3,44_ = 14.11, *p* < 0.0001), age (F_1,44_ = 10.44, *p* = 0.0023), and their interaction (F_3,44_ = 3.154, *p* = 0.0341). A post hoc test showed increased time spent in the center of the arena in young CUMS + K animals (87.10 ± 14.27 s) compared to aged counterparts (46.28 ± 21.66 s) (*p* = 0.0003), and no other age-related differences between the other mice groups (*p* > 0.05) (Figure 3B).

The use of two-way ANOVA in order to evaluate short-term memory in NORT revealed differences between treatments (F_3,44_ = 5.536, *p* = 0.0026) and age (F_1,44_ = 6.89, *p* = 0.0118), with significant interaction (F_3,44_ = 4.033, *p* = 0.0128). Post hoc tests revealed that aged CUMS + MCD mice showed an increased preference for novel objects (64.68 ± 6.21%), compared to the young CUMS + MCD group (46.61 ± 10.53%) (*p* = 0.0042). A similar difference was observed in young ketamine-treated mice with liver damage (47.68 ± 14.76%) compared to aged-treated animals (60.51 ± 6.91%), (*p* = 0.0345) (Figure 3C).

Post treatment, two-way ANOVA showed significant variances between treatments (F_3,44_ = 7.75, *p* = 0.0003) in social novelty test, but no differences between Age (F_1,44_ = 1.966, *p* = 0.1679), with significant interaction between the tested factors (F_3,44_ = 77.34, *p* < 0.0001). Regardless of ketamine administration, aged CUMS (61.76 ± 3.83%) and CUMS + K (73.46 ± 4.45%) mice exhibited increased preference for social novelty at the end of the experiment compared to young ones (38.54 ± 6.50%; respectively 40.44 ± 10.73%) (*p* < 0.0001) (Figure 3D). Irrespective of ketamine administration, an MCD diet led to decreased index in social novelty in aged CUMS + MCD (29.57 ± 2.60%) and CUMS + MCD + K (44.96 ± 3.50%) animals compared to young CUMS + MCD (61.22 ± 5.80%) (*p* < 0.0001) and CUMS + MCD + K (59.66 ± 6.92%) (*p* = 0.0002) groups (Figure 3D).

### 2.5. Acute Ketamine Treatment Can Prevent Astrogliosis and Neuronal Loss

**Figure 4:**
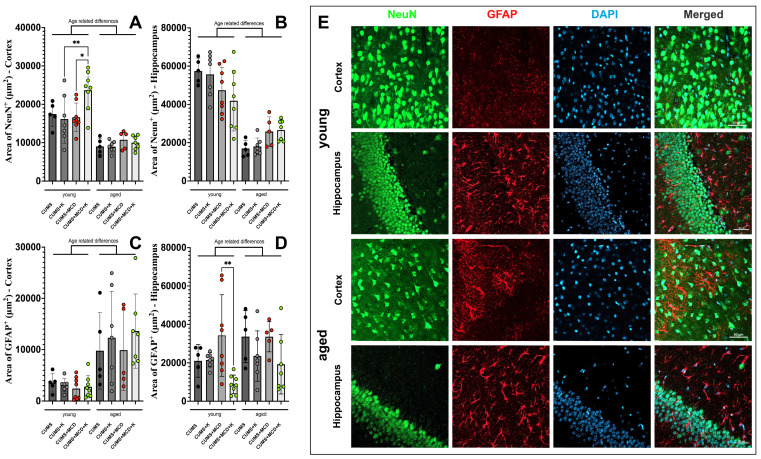
Immunohistochemical detection of NeuN-positive neurons in (**A**) the cortex revealed age-related differences, and increased NeuN+ signal area for young CUMS + MCD + K animals, compared to CUMS + K and CUMS + MCD mice. Similar age-related differences were observed in the (**B**) hippocampus, displaying a reduction in the cortical NeuN+ signal in all aged subjects compared to their younger counterparts. Age-related differences were also observed when analyzing both (**C**) cortical and (**D**) hippocampal area of GFAP-positive astrocytes, with increased signal observed in aged animals. (**E**) Neurons, astrocytes, and cell nuclei labeled with NeuN (green), GFAP (red), and DAPI (blue) for aged and young animals. The graphs show mean values ± SD, * *p* < 0.05, ** *p* < 0.01. Scale bar 50 µm.

One-way ANOVA performed in order to assess the differences in the cortical NeuN+ signal revealed differences between treatments (F_7,44_ = 13.47, *p* < 0.0001). The cortical NeuN+ signal area of the ketamine-treated young animals fed MCD and subjected to CUMS was higher (23,667 ± 5116 μm^2^), compared to CUMS + K (16,149 ± 6270 μm^2^; *p* = 0.0084) and CUMS + MCD (16,639 ± 3703 μm^2^; *p* = 0.0120) (Figure 4A). No changes were detected in the cortical NeuN+ signal of aged animals, irrespective of their hepatic condition and administration of ketamine (*p* > 0.05). At the hippocampus level, one-way ANOVA also showed variances between treatments (F_7,44_ = 16.85, *p* < 0.0001). A reduction in the cortical NeuN+ signal was observed in all aged subjects in comparison with their younger counterparts, both in the cortex (Figure 4A) and the hippocampus (Figure 4B) (Supplementary Table S3).

One-way ANOVA also revealed differences in GFAP+ signal between treatments in the cortex (F_7,44_ = 4.909, *p* = 0.0004) and the hippocampus (F_7,44_ = 3.389, *p* = 0.0056). The cortical GFAP+ signal of all animals, regardless of treatment and/or liver damage, exhibited no differences (*p* > 0.05) (Figure 4C). In the hippocampus, ketamine administration led to a reduced GFAP+ signal in young MCD-fed mice (8798 ± 4849 μm^2^), compared to untreated ones (34,186 ± 21,215 μm^2^) (*p* = 0.0056). Same tendency was observed for aged animals, but significance was not reached (*p* > 0.05) (Figure 4D). Age-related differences were observed when analyzing both cortical and hippocampal area of GFAP+ signal (Supplementary Table S4). Immunohistochemical detection of NeuN-positive neurons and GFAP-positive astrocytes in the context and hippocampus of young and aged animals is shown in Figure 4E.

**Figure 5:**
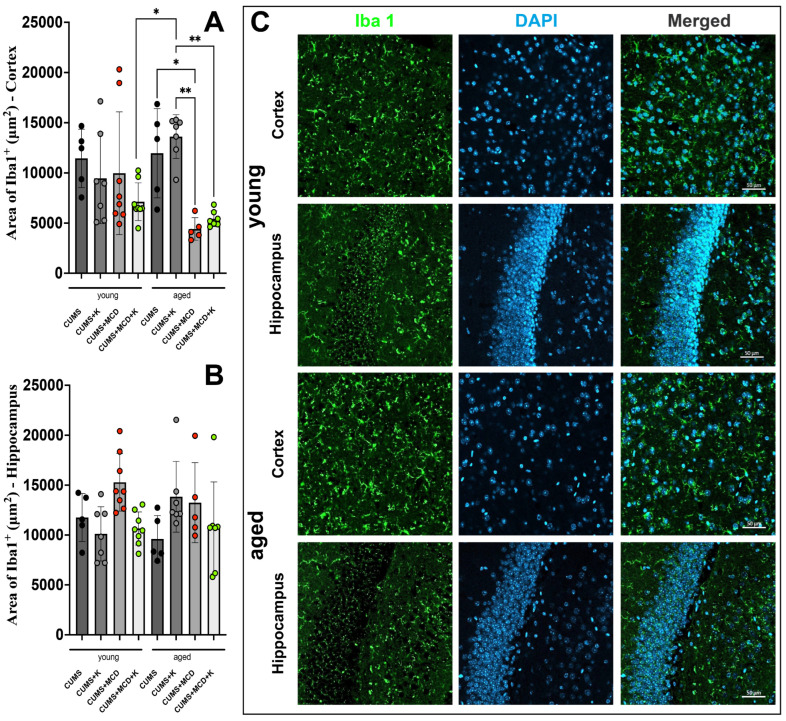
Immunohistochemical detection of Iba-1-positive microglia in (**A**) the cortex showed no differences in young animals, but decreased Iba1+ signal for all aged animals fed an MCD diet, regardless of ketamine treatment. No significant effect on the area of Iba1+ signal was observed in (**B**) the hippocampus. (**C**) Microglia and cell nuclei labeled with Iba1 (green) and DAPI (blue). The graphs show mean values ± SD, * *p* < 0.05, ** *p* < 0.01. Scale bar 50 µm.

The use of one-way ANOVA in order to evaluate the Iba1+ cortical signal showed differences between treatments (F_7,44_ = 5.091, *p* = 0.0003). The cortical Iba1+ signal of young animals, regardless of treatment and/or liver damage, exhibited no differences (*p* > 0.05) (Figure 5A). Aged animals submitted to an CUMS and MCD diet exhibited a decreased Iba1+ area (4417 ± 1120 μm^2^) compared to aged animals subjected to CUMS without liver damage (11,962 ± 4445 μm^2^) (*p* = 0.0331) (Figure 5A).

The administration of ketamine did not mitigate this decrease, with aged mice with liver damage having a lower cortical area of Iba1+ signal (4417 ± 1120 μm^2^) compared to aged mice without any liver damage and ketamine treatment (13,621 ± 2172 μm^2^) (*p* = 0.0016). This group also exhibited an increased Iba1+ signal compared to the aged CUMS + MCD + K group (5430 ± 781.2 μm^2^) (*p* = 0.0022). A solely age-related difference was found for the MCD diet between young CUMS + MCD + K (7127 ± 1882 μm^2^) and aged CUMS + K (13,621 ± 2172 μm^2^) animals (*p* = 0.0210) (Figure 5A). At the level of the hippocampus, the intervention did not demonstrate any significant effect on the area of Iba1+ signal (Figure 5B). Immunohistochemical detection of Iba-1-positive microglia in the context and hippocampus of young and aged animals is shown in Figure 5C.

## 3. Discussion

With the increase in life expectancy, healthcare professionals are faced with associated pathologies that, sometimes, need opposite therapeutic strategies. In order to better understand the molecular and cellular mechanisms of NAFLD, several animal models of NAFLD have been already developed [27]. One of the most common models used is the MCD diet [28]. Its popularity mainly comes from the liver cytological similitude to human NAFLD [29,30]. At the same time, several animal models of depression have been utilized in experimental work [31]. The CUMS model seems to lead to complex behavior changes in animals [32,33]. The main concern comes from behavioral variability generated by the dose requirements to establish the antidepressant effect of ketamine and its effect duration [34,35,36]. Selective serotonin reuptake inhibitors (SSRIs) are first-line treatments for depression [37], but ketamine has shown rapid and effective results in severe or treatment-resistant cases [38]. This study focused on ketamine’s therapeutic and toxicological effects in a model reflecting the interaction between depression and NAFLD. Since SSRIs are metabolized in the liver and may pose risks in liver disease, such as potential hepatotoxicity with drugs like sertraline and fluoxetine [39,40,41], ketamine was evaluated as an alternative in this dual-pathology context. Unlike classic antidepressants, which act slowly, ketamine targets NMDA receptors and rapidly enhances synaptic plasticity, offering faster relief [42]. For patients unresponsive to multiple antidepressants, even after augmentation, ketamine provides a distinct mechanism and a valuable alternative. While not a first-line treatment, it serves as a critical option for managing complex, resistant cases in psychiatric and neurological care.

There are several additional factors of concern observed in experimental studies, such as a reported dose-dependent effect of ketamine on neuroinflammation [43] and the severity of ketamine-induced liver toxicity [44,45]. However, there is a gap in the specialized literature regarding its utilization in murine models of depression with pre-existing liver injury. Although previous research indicates that administering ketamine in low doses (0.25, 0.50, and 1 mg/kg) did not significantly affect liver histopathology [44], we showed in the present study that a single dose of ketamine (50 mg/kg) does not worsen pre-existing liver damage, as determined by abdominal ultrasonography. Furthermore, the acute administration of ketamine did not affect the severity of liver injury caused by the diet in either young or aged mice. However, ketamine is primarily metabolized in the liver by cytochrome P450 enzymes, particularly CYP2B6 and CYP3A4, into its active metabolite, norketamine, which contributes to its therapeutic effects [46,47]. Liver dysfunction, such as that induced by the MCD diet, can significantly impair this metabolic process, potentially leading to altered plasma levels of ketamine and its metabolites. The MCD diet is associated with hepatic damage, reduced metabolic enzyme activity, and impaired liver function, all of which could affect ketamine pharmacokinetics [30,48]. These changes may result in prolonged ketamine half-life, higher systemic levels, or reduced production of norketamine, thereby altering both its therapeutic efficacy and its potential for adverse effects. Differences in ketamine’s effects between animals with and without liver injury may not solely reflect its interaction with neuroinflammatory or behavioral pathways but could also be influenced by altered drug metabolism. Future studies should incorporate measurements of plasma and tissue concentrations of ketamine and its metabolites to better elucidate these interactions.

With a majority of research studies suggesting that the effect of ketamine appears to also be dose-dependent in terms of behavioral changes, the 50 mg/kg dose was chosen as a balance between the previously 10 mg/kg dose that was shown to reverse the deficit in sucrose preference, and the 100 mg/kg dose that was proven ineffective in ameliorating sucrose preference deficits in chronically stressed mice [49]. Depression-like behavior in animal models, especially under the CUMS protocol, is assessed using well-established, validated behavioral tests. In our study, we used a standard set of such tests to confirm depressive-like states and evaluate treatment effects, in line with widely accepted preclinical practices [50,51,52,53,54,55]. We were able to show that animals treated with 50 mg/kg of ketamine exhibited decreased anhedonia, as assessed by the SPT. Interestingly, only young and not aged CUMS + MCD mice displayed increased sucrose preference after transitioning to a normal diet. Although clinical research has shown that liver damage is associated with worsening depression [56] in mice, the observed outcome may result from the MCD diet itself, as animals on this diet experience weight loss, hypermetabolism, and reduced nutrient-to-energy conversion [57], potentially leading to an increased intake of the sweet solution compared to animals fed standard chow. However, as already stated, this phenomenon was not observed in aged animals, where improvements in anhedonia were solely apparent following ketamine administration. Furthermore, at the dosage of 50 mg/kg, the antidepressant effect of ketamine persisted up to 3 weeks post-administration, a longer duration compared to previous studies where at the dose of 10 mg/kg, this effect could no longer be observed 7 days after injection [36]. In the present study, ketamine treatment and the transition to a normal diet resulted in decreased anxiety-like behavior. This might suggest that alterations in anxiety-like behavior are also dosage-dependent, taking into account that prior studies on C57BL/6 mice showed that doses ranging from 1 to 10 mg/kg may cause an increase in anxiety, as measured by a reduction in time spent in open space. Interesting, similar studies performed on Balb/c animals have reported a decrease in anxiety at 10 mg/kg of ketamine, as seen by the increased time spent in the open space [58]. These contradictory results suggest that future studies should consider species variability when assessing the anxiolytic effects of ketamine. Moreover, differences in aged and young animals, such as young CUMS + K animals, exhibited decreased anxiety and increased sucrose preference, compared to their aged counterparts, which also indicates that age should be strongly considered in future research.

While previous studies have reported that acute administration of low-dose ketamine (10 mg/kg) may enhance the NOR preference index [49,59] and that a high dose of ketamine (100 mg/kg) impairs short-term object recognition memory [49], here, we showed that a 50 mg/kg does of ketamine yields no improvement in memory assessment, irrespective of hepatic status or age. Moreover, it seems that the administration of the 5 weeks of MCD diet led to decreased preference for the novel object, regardless of the ketamine treatment. This difference was especially observed in young animals, in which liver damage more obviously affected the recognition index. This is surprising as previous work has shown that 4 weeks of MCD diet did not affect NOR performance in young animals [60]. Regarding social preference, previous studies indicate that ketamine, at the dose of 10–15 mg/kg, appears to decrease the overall social interaction time [61], while concurrently mitigating social avoidance in defeated mice at 20 mg/kg [62]. Our findings revealed that in aged animals, 50 mg/kg of ketamine enhanced the social preference index, irrespective of diet. In contrast, young CUMS animals on the MCD diet exhibited a heightened preference for social novelty, independent of ketamine treatment. It is well recognized that various diets can profoundly influence social behavior in rodents. A high-fat diet has been shown to decrease social interaction [63,64] and elevate anxiety-like behaviors [65,66], while a ketogenic diet may promote social exploration [67,68,69] and reduce anxiety [70]. Here, we were able to show that MCD diet may also influence the social behavior in young but not aged C57/BL6 mice.

The observed increase in the NeuN+ signal in young animals subjected to the MCD diet and ketamine treatment highlights the potentially synergistic effect of ketamine on neuronal preservation or activation in the context of liver injury and chronic stress. Previous research has suggested that the MCD diet can reduce the cortical area of NeuN+ signal, likely due to neuroinflammation or neuronal damage associated with liver dysfunction and metabolic stress [60], However, ketamine’s known ability to activate adult-born immature granule neurons could counteract these effects, leading to an increase in NeuN+ labeling [71]. Ketamine may promote neuronal survival or enhance neurogenesis, particularly in younger animals, even under the combined stress of a CUMS and MCD diet. Furthermore, age-related differences in NeuN+ signal imply that younger brains may be more responsive to ketamine’s neuroprotective effects, a phenomenon that warrants further investigation. These results underscore ketamine’s potential not only as an antidepressant, but also as a modulator of neuronal health in conditions of systemic stress and injury.

Our findings align with previous research demonstrating that ketamine may exert anti-activating effects on astrocytes [43], contributing to its potential neuroprotective and anti-inflammatory properties. Astrocyte activation, marked by increased expression of GFAP, is a hallmark of neuroinflammation [72] and is often observed in various neurological and psychiatric disorders, including depression [73]. Ketamine has been shown to modulate astrocyte activity, likely through its interaction with NMDA receptors and downstream signaling pathways, including the suppression of pro-inflammatory cytokine release and regulation of glutamate metabolism [74,75,76]. Specifically, acute ketamine treatment has been reported to significantly reduce the number of GFAP+ cells in the hippocampus [77], a brain region critically involved in mood regulation and cognitive function [78]. This reduction in astrocytic activation may help mitigate the neuroinflammatory responses associated with chronic stress or systemic insults, such as liver injury. In our study, the observed decrease in GFAP+ signal in the hippocampus following ketamine administration supports the hypothesis that ketamine can attenuate astrocytic activation in pathological conditions. This effect may play a crucial role in the rapid alleviation of depressive-like behaviors as astrocyte dysfunction and heightened neuroinflammation have been implicated in the pathophysiology of depression. The ability to reduce astrocytic activation could contribute to ketamine’s rapid and sustained antidepressant effects, particularly in conditions characterized by heightened neuroinflammatory states [79,80]. Future research should investigate the molecular mechanisms underlying this effect, including the role of specific astrocytic signaling pathways and their interactions with ketamine’s pharmacodynamics, to fully understand the therapeutic implications of these findings. Although previous studies have shown that acute ketamine treatment reduces the number of Iba1^+^ glial cells [77], in our animals, a reduction in Iba1^+^ signal was only observed in MCD fed animals, with the additional administration of ketamine having no effect on Iba1^+^ cellular response. These differences were observed in aged animals, contrasting with previous research on young mice, which found no differences in the cortical area occupied by Iba1^+^ cells after MCD diet administration [60]. Future research should consider animals’ age when evaluating the inflammatory response to both ketamine treatment and the MCD diet.

The present study has several limitations. This study is that it does not incorporate the complex and multifactorial mechanisms involved in the co-development of depression and NAFLD in humans. Key pathological contributors such as genetic predisposition, insulin resistance, and immune-mediated pathways were not addressed and should be considered in future studies to enhance translational relevance. We used a relatively high dose of ketamine (50 mg/kg) but did not explore the effects of different dosages across various behavioral and physiological parameters. Also, the fact that the study focuses on the effects of acute, single-dose administration of ketamine poses a severe limitation on the legitimate question regarding repetitive and long-term administration of ketamine in NAFLD patients. Chronic administration protocols were not examined. The main reason is that clinical studies have shown a high compliance to NAFLD treatment [81,82], but this alone does not justify a potential chronic ketamine administration. The study uses C57BL/6 mice, and while these are a common model, there may be strain-specific responses to both the MCD diet and ketamine treatment. The study primarily focuses on GFAP^+^ and Iba1^+^ signals as markers of glia activation and neuroinflammation, but additional markers and a broader analysis of neuroinflammatory pathways would enhance the understanding of ketamine’s effects on neuroinflammation in animals with liver damage. With the purpose of any basic research to generate translational research, here we also suggest that any such endives must encompass particularities related to alcohol consumption, which, even in moderate amounts, appears to induce further liver alterations in individuals with depression and anxiety disorders [83,84].

It is important to emphasize that this study is primarily investigative in nature. It is not intended to serve as direct clinical guidance, nor should it dictate medical decision-making in current practice. Rather, our results should be viewed as a foundation for future translational research, particularly in light of the growing clinical burden posed by coexisting chronic diseases such as depression and NAFLD. Given ketamine’s unique mechanism of action and rapid onset of antidepressant effects, our findings suggest it may represent a valuable therapeutic option in complex cases, especially where standard antidepressants have failed. However, careful clinical evaluation, rigorous trials, and interdisciplinary collaboration will be essential to determine its appropriate place in treatment algorithms.

Prospective studies should aim to compare the efficacy and safety of ketamine with first-line antidepressants, explore long-term effects and repeated administration protocols, and incorporate broader pathophysiological mechanisms. Additionally, it is crucial to investigate sex-specific responses in the context of depression and its treatment, as hormonal factors have been shown to significantly influence the vulnerability to depression. Notably, female mice exhibit heightened sensitivity to ketamine’s antidepressant effects compared to male mice [26]. This suggests that gender-related differences in ketamine response may play an important role in determining the drug’s efficacy and mechanisms of action. In our current study, we limited our behavioral testing to male mice and utilized a single administration of ketamine. While this approach allowed us to focus on the initial response to treatment, we acknowledge that the exclusion of female subjects and the use of only one dose of ketamine may restrict the generalizability of our findings regarding sex differences in both depression vulnerability and treatment response. Finally, it is important to translate findings into clinical trials, particularly in treatment-resistant depression cohorts with metabolic comorbidities, to evaluate safety, efficacy, and tolerability in a real-world setting.

## 4. Materials and Methods

### 4.1. Experimental Animals

The study was performed on male C57BL/6N mice, housed in individual ventilated cages on a 12 h light/dark cycle at 20 °C. The experimental animals (16–18-week-old (*n* = 28) and 70–72-week-old (*n* = 24)) were allowed a 72 h period to acclimatize to the new laboratory conditions before the start of any procedures. The mice were obtained from the Animal Facility of the University of Medicine and Pharmacy of Craiova. All experimental protocols and animal care were approved by the Committee for Experimental Animals Wellbeing of the University of Medicine and Pharmacy of Craiova (approval nos. 2.13 from 29 October 2020 and 2.1 from 10 November 2022).

### 4.2. Induction of Depressive-like Behavior and Non-Alcoholic Fatty Liver Disease/Non-Alcoholic Steatohepatitis

All animals were exposed to the chronic unpredictable mild stress (CUMS) procedure as previously described [85]. Although the term “mild” is part of the CUMS protocol’s name, it describes the individual stressors, not the overall effect. When applied unpredictably over time, the protocol consistently produces a strong and clinically relevant depressive-like state in rodents [86]. Shortly, within a 28-day period, the animals were repeatedly exposed to seven different mild and unpredictable stressors, one each day, with no stressor repeated earlier than 3 days. During this period, all mice were individually housed as recommended [87].

The non-alcoholic, non-viral hepatitis was induced over a period of 5 weeks, as previously detailed [88], using a methionine/choline chloride-deficient (MCD) diet (MP Biomedicals, Eschwege, Germany).

The CUMS protocol is a widely accepted model for inducing depression-like behavior in rodents and is commonly used the antidepressant effects across a variety of pharmacological and natural compounds [86,89,90,91,92]. Similarly, the MCD diet reliably induces NAFLD in animals, mimicking key aspects of human liver disease [28,93]. Based on prior comparative research from our lab, combining CUMS with the MCD diet best replicates the interaction between depression and liver injury [85].

After 5 weeks of MCD diet, the treatment groups received a single i.p. ketamine (K) injection (50 mg/kg) in a 150 µL volume, while the others a single i.p. saline injection (150 µL). Both young and old animals were randomly assigned to the CUMS (*n* = 10), CUMS + K (*n* = 14), CUMS + MCD (*n* = 13) or CUMS + MCD + K (*n* = 15) groups before the start of any procedures.

### 4.3. Clinical Evaluation and Behavior Testing

All mice were tested before the start of any procedure in order to establish a baseline. To minimize potential effects from behavioral testing, we allowed an additional week of habituation before starting the experimental protocols (Figure 1A). This ensured any residual effects from baseline testing or abdominal ultrasonography had subsided, and the first weighing was conducted at the beginning of the protocols for accurate, consistent measurements. Subsequently, the body weight of the mice was measured weekly throughout the duration of the experiment.

Following 4 weeks of exposure to CUMS and MCD diet, all mice underwent retesting to assess the effects of the experiments on their behavior, followed by the administration of ketamine treatment. Subsequently, all mice were transitioned to a normal diet and reevaluated 2 weeks after treatment (Figure 1A). Prior to each behavioral test, the animals were allowed one hour to acclimate to the testing environment. Following each trial, the testing surface was cleaned using 75% ethanol to eliminate odors.

The sucrose preference test (SPT), open field test (OFT), novel object recognition test (NORT) and Crawley’s sociability and preference for social novelty test were performed on different days, but at the same hour.

SPT was performed as previously described [85] in order to assess anhedonia [94]. Briefly, the animals were allowed 4 days of habituation with 2 drinking bottles, one containing 2% sucrose solution and the other tap water. Before the test, animals were deprived of food and water for 12 h. After this interval, the animals were given free access to both for 24 h. The position of the two bottles was changed 12 h after the start of the procedure in order to reduce any side bias. At the end of the procedure the volume of sucrose and water consumed were recorded and the animals’ affinity to sucrose was calculated. Sucrose preference was displayed as the percentage of the volume of sucrose consumption over the total fluid consumption during the 24 h testing period.

OFT was conducted according to already published protocols [60,85]. All the trials were carried out in an open arena (50 cm (length) × 33 cm (width) × 15 cm (height)). Animals were placed in the center of the arena and observed for 10 min. The duration spent by the mouse in the central squares versus the peripheral squares was utilized as a metric for assessing anxiogenic behavior. All parameters analyzed by the OFT were automatically generated (EthoVision XT 17, Noldus Technology, Wageningen, The Netherlands).

NORT was conducted as previously described [85,88] to assess short-term memory [95,96]. The animals were allowed to freely explore an open field-like arena with two identical objects for 6 min. After a 1 h break in their normal cage, they returned to the arena for another 6 min, but one object was replaced. Using EthoVision XT 17, the preference index was calculated based on the time spent exploring the new object compared to both objects.

The Crawley’s sociability and preference for social novelty test allows the quantification of social behavior and the preference for social novelty [97]. A three-chamber box was used to perform this test. The chambers are divided by clear glass walls and have openings that allow free access to all chambers [97]. Four sessions were performed for each animal: two habituation and two testing sessions. For habituation, the tested mouse was placed in the middle chamber for 10 min, with the right and left compartments isolated. Then, the animal was allowed to explore all three chambers for another 10 min. Within the left and right chambers, two identical, wire cup-like containers, large enough to hold a single mouse, were placed. Within one of the containers, a “Stranger 1” mouse was placed with the same background, gender, weight, and age, without any prior contact with the tested mouse. The tested animal was allowed to freely explore for an additional 10 min. In the second session, a “Stranger 2” animal was placed in the empty wire cup-like container. The subject was recorded for an additional 10 min. Using EthoVision XT 17, the preference for social novelty was determined for each mouse.

### 4.4. Abdominal Ultrasonography

Hepatic ultrasound assessments were conducted utilizing a S12-4 plane probe and a Philips CX50 ultrasound machine (Philips Healthcare, Amsterdam, The Netherlands). Parameters such as parenchymal echotexture, nodular presence, and the liver border surface were utilized to determine the severity score, as previously described [85]. Throughout the procedure, animals were anesthetized using a mixture of 1.5% isoflurane, 49% O_2_, and 49% N_2_O administered via inhalation.

### 4.5. Histopathology and Immunohistochemistry

After being deeply anesthetized, the animals were intracardially perfused with 5 mL saline, followed by 5 mL 4% paraformaldehyde. The brains were left overnight in 4% PFA at 4 °C, in order to minimize microglia activation [98]. All immunohistochemistry was performed on 35-μm-thick coronal sections cut into a bath of 0.1 M phosphate-buffered saline (PBS). Shortly, sections were blocked with 0.5% Triton X-100, 5% horse serum in PBS for 1 h at room temperature, followed by overnight incubation (4 °C) with the primary antibodies: mouse NeuN monoclonal (Invitrogen, Waltham, MA, USA, MA5-33103, 1:500), rabbit anti-Iba1 (Wako, Osaka, Japan, 019-19741, 1:1000), and rabbit anti-GFAP polyclonal (Dako, Carpinteria, CA, USA, Z0334, 1:1000). After washing, sections were incubated for 2 h at room temperature, in the dark, with secondary antibodies: Alexa Fluor 488 donkey anti-mouse (Invitrogen, Waltham, MA, USA, A21202, 1:1000) and Alexa Fluor 647 donkey anti-rabbit (Invitrogen, Waltham, MA, USA, A31573, 1:1000). Brain sections were then mounted and covered with Fluoromount-G with DAPI (ThermoScientific, Waltham, MA, USA, 00-4959-52).

### 4.6. Image Analysis

For analysis, Z-stack images of cortex and hippocampus were taken using the 20× objective of a Zeiss LSM 900 Airyscan 2 confocal microscope (Jena, Germany) and Zen 3.5 software. The area of cortical and hippocampal NeuN, GFAP, and Iba1 were determined for each animal using Fiji 2.0.0 and Image-Pro Plus 11. Initially, the acquired images were processed in Fiji Image J to separate the channels, allowing independent analysis of each cell type. Each channel was converted to a black and white image, and the intensity, brightness, and contrast parameters were adjusted to optimize the visibility of the specific signal, to distinguish the signal from background noise, and to ensure accurate quantification [99,100,101,102]. The image processed in Fiji Image J 2.0.0 was imported into Image ProPlus 11 software (Media Cybernetics, Bethesda, MD, USA) for analysis. The acquisition parameters were calibrated to ensure accurate measurements. After calibration, the signal quantification function was used, and the software measured the area and signal intensity for each marker in the cortical and hippocampal regions. For each animal, the signal was quantified from four different images of the cortex and four of the hippocampus. The values obtained from the quantification of the four images were used to calculate the arithmetic mean of the signal for each marker. This average represented the final signal value for each animal, both in the cortex and hippocampus.

### 4.7. Statistical Analysis

GraphPad Prism 10 and Microsoft Excel 2016 were used for statistical analysis. Differences in means among the groups were analyzed using one or two-way ANOVA (Tukey’s multiple comparisons test), with repeated measurements and Geisser–Greenhouse correction, after the data set passed normality testing (Shapiro–Wilk test and Kolmogorov–Smirnov test), and the Kruskal–Wallis test (Dunn’s multiple comparations test) for non-parametric data. For ANOVA test, sessions (baseline and weekly results) were used as a within-factor, and Treatments (CUMS, CUMS + K, CUMS + MCD, CUMS + MCD + K) were considered as a between-factor. For the comparation between young and aged animals, Treatments were used as a within-factor, and age was considered as a between-factor. All figures show mean value and standard deviation (SD), and the statistical significance was displayed as follows: * *p* < 0.05, ** *p* < 0.01, *** *p* < 0.001, **** *p* < 0.0001 displayed differences between treatments, and # *p* < 0.05, ## *p* < 0.01, ### *p* < 0.001, #### *p* < 0.0001 displayed differences between sessions.

## 5. Conclusions

Single-dose administration of 50 mg/kg ketamine does not exacerbate the severity of liver injury. Furthermore, ketamine effectively alleviates anhedonia and anxiety-like behavior, irrespective of liver damage, suggesting its broad therapeutic potential in addressing mood-related symptoms. However, while ketamine does not enhance memory recognition, it shows promise in improving sociability, particularly in aged subjects. Interestingly, our findings indicate that ketamine may be more efficacious in young animals compared to aged counterparts. Additionally, acute ketamine treatment appears to lead to the inhibition of activity in cortical and hippocampal immunohistochemical detection. Overall, this study underscores the multifaceted effects of ketamine and highlights its therapeutic versatility, while also suggesting differential responses based on age.

## Data Availability

The data presented in this study are available upon request from the corresponding authors.

## Supplementary Materials

The following supporting information can be downloaded at: https://www.mdpi.com/article/10.3390/ijms26083558/s1.

## Abbreviations

The following abbreviations are used in this manuscript:

CUMS	Chronic unpredictable mild stress
GFAP	Glial fibrillary acidic protein
Iba 1	Ionized calcium binding adaptor molecule 1
K	Ketamine
KILI	Ketamine-induced liver injury
MCD	Methionine–choline deficient
NAFLD	Non-alcoholic fatty liver disease
NeuN	Neuronal nuclei
NORT	Novel object recognition test
OFT	Open field test
PBS	Phosphate-buffered saline
PFA	Paraformaldehyde
SD	Standard deviation
SPT	Sucrose preference test
SSRI’s	Selective serotonin reuptake inhibitors
