# Supplementary material for: The Behavioral and Neuroinflammatory Impact of Ketamine in a Murine Model of Depression and Liver Damage
Source: Int J Mol Sci. 2025 Apr 10;26(8):3558. doi: 10.3390/ijms26083558 (PMC12027082; doi:10.3390/ijms26083558)
Supplement: Supplementary file 1 [file ijms-26-03558-s001.zip › ijms-3553764-supplementary.pdf]

Supplementary Material

1 **Supplementary Table S1.** SPT weekly differences according to two-way ANOVA, post-hoc test

SUCROSE PREFERENCE TEST

Tukey's multiple comparations test

YOUNG ANIMALS	Mean Diff.	95,00% CI of diff	Summary	Adjusted P Value
CUMS				
Baseline vs. Week 5	10,70	3,911 to 17,48	*	0,0108
Baseline vs. Week 8	9,479	-1,584 to 20,54	ns	0,0793
Week 5 vs. Week 8	-1,217	-5,517 to 3,083	ns	0,6104
CUMS+K				
Baseline vs. Week 5	11,04	7,642 to 14,45	***	0,0001
Baseline vs. Week 8	-3,425	-7,918 to 1,069	ns	0,1254
Week 5 vs. Week 8	-14,47	-17,61 to -11,33	****	<0,0001
CUMS+MCD				
Baseline vs. Week 5	10,90	9,105 to 12,70	****	<0,0001
Baseline vs. Week 8	5,884	2,966 to 8,801	**	0,0014
Week 5 vs. Week 8	-5,018	-8,376 to -1,661	**	0,0077
CUMS+MCD+K				
Baseline vs. Week 5	8,313	4,856 to 11,77	***	0,0005
Baseline vs. Week 8	-0,6583	-2,088 to 0,7716	ns	0,4118
Week 5 vs. Week 8	-8,971	-11,39 to -6,547	****	<0,0001

Row statistics

	CUMS			CUMS+K			CUMS+MCD			CUMS+MCD+K		
	Mean	SD	N	Mean	SD	N	Mean	SD	N	Mean	SD	N
Baseline	85.532	5.685	5	86.759	3.989	7	89.991	1.671	8	87.074	1.547	8
Week 5	74.835	1.979	5	75.715	1.491	7	79.089	2.624	8	78.761	3.330	8
Week 8	76.053	1.995	5	90.184	2.611	7	84.107	2.803	8	87.732	1.459	8

Tukey's multiple comparations test

AGED ANIMALS	Mean Diff.	95,00% CI of diff	Summary	Adjusted P Value
CUMS				
Baseline vs. Week 5	6,025	2,849 to 9,200	**	0,0055
Baseline vs. Week 8	4,100	-0,09739 to 8,297	ns	0,0538
Week 5 vs. Week 8	-1,925	-7,323 to 3,474	ns	0,4799
CUMS+K				
Baseline vs. Week 5	5,172	2,343 to 8,000	**	0,0033
Baseline vs. Week 8	-6,355	-9,529 to -3,181	**	0,0021
Week 5 vs. Week 8	-11,53	-12,61 to -10,44	****	<0,0001
CUMS+MCD				
Baseline vs. Week 5	0,3053	-9,505 to 10,12	ns	0,9933
Baseline vs. Week 8	-0,6972	-6,962 to 5,567	ns	0,9186
Week 5 vs. Week 8	-1,003	-6,336 to 4,331	ns	0,7922
CUMS+MCD+K				
Baseline vs. Week 5	-1,216	-10,61 to 8,184	ns	0,9180
Baseline vs. Week 8	-9,305	-13,93 to -4,677	**	0,0020
Week 5 vs. Week 8	-8,089	-14,15 to -2,029	*	0,0151

Row statistics

	CUMS			CUMS+K			CUMS+MCD			CUMS+MCD+K		
	Mean	SD	N	Mean	SD	N	Mean	SD	N	Mean	SD	N
Baseline	79.619	1.837	5	79.579	3.102	7	80.481	4.154	5	79.599	5.624	7
Week 5	73.594	1.907	5	74.408	2.300	7	80.175	4.473	5	80.814	5.387	7
Week 8	75.519	1.949	5	85.934	2.564	7	81.178	2.492	5	88.904	2.524	7

2 **Supplementary Table S2.** OFT weekly differences between young animals and aged animals in Crawley's sociability test according to two-way ANOVA, post-hoc test

OPEN FIELD TEST

Tukey's multiple comparations test

YOUNG ANIMALS	Mean Diff.	95,00% CI of diff	Summary	Adjusted P Value
CUMS				
Baseline vs. Week 5	43,07	25,28 to 60,86	**	0,0022
Baseline vs. Week 8	42,39	25,18 to 59,59	**	0,0021
Week 5 vs. Week 8	-0,6786	-19,27 to 17,91	ns	0,9907
CUMS+K				
Baseline vs. Week 5	23,54	-1,867 to 48,94	ns	0,0662
Baseline vs. Week 8	-19,00	-45,68 to 7,691	ns	0,1528
Week 5 vs. Week 8	-42,53	-52,16 to -32,91	****	<0,0001
CUMS+MCD				
Baseline vs. Week 5	14,68	3,516 to 25,84	*	0,0147
Baseline vs. Week 8	-18,42	-38,49 to 1,655	ns	0,0697
Week 5 vs. Week 8	-33,09	-47,42 to -18,76	***	0,0006
CUMS+MCD+K				
Baseline vs. Week 5	22,41	9,796 to 35,02	**	0,0030
Baseline vs. Week 8	1,688	-6,396 to 9,772	ns	0,8168
Week 5 vs. Week 8	-20,72	-34,08 to -7,354	**	0,0064

Row statistics

	CUMS			CUMS+K			CUMS+MCD			CUMS+MCD+K		
	Mean	SD	N	Mean	SD	N	Mean	SD	N	Mean	SD	N
Baseline	88.080	10.224	5	68.114	12.519	7	71.145	9.754	8	75.820	13.324	8
Week 5	45.013	9.155	5	44.575	10.997	7	56.469	4.847	8	53.414	12.178	8
Week 8	45.692	3.826	5	87.110	14.279	7	89.562	14.787	8	74.132	8.202	8

CRAWLEY'S SOCIABILITY TEST

Tukey's multiple comparations test

AGED ANIMALS	Mean Diff.	95,00% CI of diff	Summary	Adjusted P Value
CUMS				
Baseline vs. Week 5	-8,483	-15,31 to -1,658	*	0,0247
Baseline vs. Week 8	-10,57	-23,64 to 2,496	ns	0,0933
Week 5 vs. Week 8	-2,087	-11,75 to 7,572	ns	0,7390
CUMS+K				
Baseline vs. Week 5	-8,450	-26,87 to 9,972	ns	0,3953
Baseline vs. Week 8	-21,11	-34,78 to -7,432	**	0,0077
Week 5 vs. Week 8	-12,66	-19,40 to -5,917	**	0,0029
CUMS+MCD				
Baseline vs. Week 5	30,34	18,66 to 42,02	**	0,0017
Baseline vs. Week 8	31,05	17,25 to 44,85	**	0,0029
Week 5 vs. Week 8	0,7106	-5,055 to 6,476	ns	0,9016
CUMS+MCD+K				
Baseline vs. Week 5	27,97	6,698 to 49,25	*	0,0161
Baseline vs. Week 8	12,81	-8,177 to 33,79	ns	0,2264
Week 5 vs. Week 8	-15,16	-22,52 to -7,808	**	0,0018

Row statistics

	CUMS			CUMS+K			CUMS+MCD			CUMS+MCD+K		
	Mean	SD	N	Mean	SD	N	Mean	SD	N	Mean	SD	N
Baseline	51.191	5.608	5	52.360	8.591	7	60.623	8.984	5	57.777	19.782	7
Week 5	59.674	3.247	5	60.810	8.842	7	30.283	1.978	5	29.805	4.405	7
Week 8	61.761	3.830	5	73.468	4.455	7	29.572	2.601	5	44.969	3.509	7

Supplementary Table S3. Aged-related differences according to one-way ANOVA in cortical and hippocampal $NeuN^+$ signal

NeuN+ signal

CORTEX

Ordinary one-way ANOVA	**Mean Diff.**	**95,00% CI of diff**	**Summary**	**Adjusted P Value**
CUMS young vs. CUMS aged	8401	802,1 to 15999	*	0,0210
CUMS young vs. CUMS+K aged	8469	1434 to 15504	**	0,0088
CUMS young vs. CUMS+MCD+K aged	7462	426,8 to 14497	*	0,0306
CUMS aged vs. CUMS+MCD young	-7593	-14443 to -743,8	*	0,0205
CUMS aged vs. CUMS+K young	-7103	-14138 to -68,38	*	0,0463
CUMS aged vs. CUMS+MCD+K young	-14621	-21470 to -7772	****	<0,0001
CUMS+MCD young vs. CUMS+K aged	7661	1443 to 13879	**	0,0068
CUMS+MCD young vs. CUMS+MCD+K aged	6654	436,1 to 12872	*	0,0283
CUMS+MCD aged vs. CUMS+MCD+K young	-12887	-19736 to -6037	****	<0,0001
CUMS+K young vs. CUMS+K aged	7171	749,3 to 13593	*	0,0191
CUMS+K aged vs. CUMS+MCD+K young	-14689	-20907 to -8471	****	<0,0001
CUMS+MCD+K young vs. CUMS+MCD+K aged	13682	7464 to 19900	****	<0,0001

Descriptive statistics	CUMS young	CUMS aged	CUMS+MCD young	CUMS+MCD aged	CUMS+K young	CUMS+K aged	CUMS+MCD+K young	CUMS+MCD+K aged
Minimum	12583	6518	11001	7968	8128	6624	13869	7365
Maximum	20883	11781	22523	12977	26235	10976	29572	12054
Range	8300	5264	11522	5009	18107	4352	15703	4689
Mean	17447	9046	16639	10780	16149	8978	23667	9985
Std. Deviation	3179	2098	3703	2360	6270	1434	5116	1823
Std. Error of Mean	1422	938,3	1309	1055	2370	542,1	1809	689,1

HIPPOCAMPUS

Ordinary one-way ANOVA	**Mean Diff.**	**95,00% CI of diff**	**Summary**	**Adjusted P Value**
CUMS young vs. CUMS aged	40493	20619 to 60367	****	<0,0001
CUMS young vs. CUMS+MCD aged	31638	11764 to 51512	***	0,0002
CUMS young vs. CUMS+K aged	39410	21010 to 57809	****	<0,0001
CUMS young vs. CUMS+MCD+K aged	30846	12446 to 49246	****	<0,0001
CUMS aged vs. CUMS+MCD young	-30470	-48384 to -12556	****	<0,0001
CUMS aged vs. CUMS+K young	-38737	-57137 to -20338	****	<0,0001
CUMS aged vs. CUMS+MCD+K young	-24962	-42877 to -7048	**	0,0015
CUMS+MCD young vs. CUMS+MCD aged	21615	3701 to 39529	**	0,0086
CUMS+MCD young vs. CUMS+K aged	29387	13123 to 45650	****	<0,0001
CUMS+MCD young vs. CUMS+MCD+K aged	20823	4559 to 37086	**	0,0043
CUMS+MCD aged vs. CUMS+K young	-29882	-48282 to -11483	***	0,0001
CUMS+K young vs. CUMS+K aged	37654	20857 to 54450	****	<0,0001
CUMS+K young vs. CUMS+MCD+K aged	29090	12293 to 45887	****	<0,0001
CUMS+K aged vs. CUMS+MCD+K young	-23879	-40142 to -7615	***	0,0007

Descriptive statistics	CUMS young	CUMS aged	CUMS+MCD young	CUMS+MCD aged	CUMS+K young	CUMS+K aged	CUMS+MCD+K young	CUMS+MCD+K aged
Minimum	49835	12752	32348	17964	38447	13823	21983	20585
Maximum	65204	22816	62573	36017	67398	26611	67462	33074
Range	15369	10064	30225	18054	28951	12787	45479	12489
Mean	57449	16955	47426	25810	55693	18039	41918	26603
Std. Deviation	6441	4165	12018	7732	11283	4471	15803	5422
Std. Error of Mean	2880	1863	4249	3458	4264	1690	5587	2049

Supplementary Table S4. Aged-related differences according to one-way ANOVA in cortical and hippocampal $GFAP^+$ signal

GFAP+ signal

CORTEX

Ordinary one-way ANOVA	**Mean Diff.**	**95,00% CI of diff**	**Summary**	**Adjusted P Value**
CUMS+MCD young vs. CUMS+K aged	-9885	-19033 to -736,1	*	0,0260
CUMS+MCD young vs. CUMS+MCD+K aged	-11187	-20336 to -2039	**	0,0074
CUMS+K young vs. CUMS+MCD+K aged	-10542	-19990 to -1093	*	0,0193
CUMS+K aged vs. CUMS+MCD+K young	9546	397,6 to 18695	*	0,0353
CUMS+MCD+K young vs. CUMS+MCD+K aged	-10849	-19997 to -1700	*	0,0104

Descriptive statistics	CUMS young	CUMS aged	CUMS+MCD young	CUMS+MCD aged	CUMS+K young	CUMS+K aged	CUMS+MCD+K young	CUMS+MCD+K aged
Minimum	1206	3232	220,0	2877	1168	1908	690,4	7337
Maximum	6182	21090	5590	18770	5379	24924	7247	27885
Range	4976	17857	5370	15893	4210	23016	6557	20547
Mean	3571	9772	2442	9897	3088	12327	2781	13630
Std. Deviation	1788	7440	2177	7807	1275	9056	2160	7223
Std. Error of Mean	799,6	3327	769,5	3491	482,1	3423	763,6	2730

HIPPOCAMPUS

Ordinary one-way ANOVA	**Mean Diff.**	**95,00% CI of diff**	**Summary**	**Adjusted P Value**
CUMS aged vs. CUMS+MCD+K young	24837	1745 to 47929	*	0,0271
CUMS+MCD aged vs. CUMS+MCD+K young	24804	1712 to 47896	*	0,0274

Descriptive statistics	CUMS young	CUMS aged	CUMS+MCD young	CUMS+MCD aged	CUMS+K young	CUMS+K aged	CUMS+MCD+K young	CUMS+MCD+K aged
Minimum	7648	17118	8911	21349	14843	5781	2685	6221
Maximum	27815	48093	65540	42647	25960	46869	16746	48419
Range	20167	30975	56629	21298	11117	41088	14061	42198
Mean	21011	33635	34186	33602	21520	23520	8798	19276
Std. Deviation	8502	13509	21215	7946	3642	13080	4849	15499
Std. Error of Mean	3802	6041	7501	3554	1377	4944	1714	5858
